# Regulation of Ovarian Cancer Stem Cells or Tumor-Initiating Cells

**DOI:** 10.3390/ijms14046624

**Published:** 2013-03-25

**Authors:** Mi Jeong Kwon, Young Kee Shin

**Affiliations:** 1 College of Pharmacy, Kyungpook National University, 80 Daehak-ro, Buk-gu, Daegu 702-701, Korea; 2 Research Institute of Pharmaceutical Sciences, College of Pharmacy, Kyungpook National University, 80 Daehak-ro, Buk-gu, Daegu 702-701, Korea; 3 Laboratory of Molecular Pathology and Cancer Genomics, College of Pharmacy, Seoul National University, 1 Gwanak-ro, Gwanak-gu, Seoul 151-742, Korea; 4 Research Institute of Pharmaceutical Sciences, College of Pharmacy, Seoul National University, 1 Gwanak-ro, Gwanak-gu, Seoul 151-742, Korea; 5 Advanced Institutes of Convergence Technology, Suwon, Gyeonggi-do 443-270, Korea

**Keywords:** cancer stem cell or tumor-initiating cells (CSC/TICs), chemoresistance, microRNA, ovarian cancer, recurrence, tumor microenvironment

## Abstract

Cancer stem cells or tumor-initiating cells (CSC/TICs), which can undergo self-renewal and differentiation, are thought to play critical roles in tumorigenesis, therapy resistance, tumor recurrence and metastasis. Tumor recurrence and chemoresistance are major causes of poor survival rates of ovarian cancer patients, which may be due in part to the existence of CSC/TICs. Therefore, elucidating the molecular mechanisms responsible for the ovarian CSC/TICs is required to develop a cure for this malignancy. Recent studies have indicated that the properties of CSC/TICs can be regulated by microRNAs, genes and signaling pathways which also function in normal stem cells. Moreover, emerging evidence suggests that the tumor microenvironments surrounding CSC/TICs are crucial for the maintenance of these cells. Similarly, efforts are now being made to unravel the mechanism involved in the regulation of ovarian CSC/TICs, although much work is still needed. This review considers recent advances in identifying the genes and pathways involved in the regulation of ovarian CSC/TICs. Furthermore, current approaches targeting ovarian CSC/TICs are described. Targeting both CSC/TICs and bulk tumor cells is suggested as a more effective approach to eliminating ovarian tumors. Better understanding of the regulation of ovarian CSC/TICs might facilitate the development of improved therapeutic strategies for recurrent ovarian cancer.

## 1. Introduction

Although high response rates to initial treatments, including surgical debulking and chemotherapy, have been achieved in patients with ovarian cancer, this disease remains the most lethal gynecological malignancy [1]. The high mortality rates of ovarian cancer patients is mainly due to recurrent and chemoresistant ovarian tumors. Most ovarian cancer patients that respond to initial therapy subsequently relapse within 5 years [2]. Therefore, the mechanisms responsible for the recurrence of ovarian cancer need to be elucidated to develop a cure for this malignancy. The Cancer Genome Atlas (TCGA) published the results of an integrated analysis of hundreds of high-grade serous ovarian cancers, which has led to a more comprehensive understanding of ovarian tumorigenesis [3]. Using high-throughput technologies, including microarray and next generation sequencing, these analyses identified genomic and epigenomic aberrations that can affect the clinical outcomes of patients. On the basis of these results, novel approaches for the development of targeted therapies for ovarian cancer have been identified. However, the data from the TCGA study was mainly obtained from genomic and epigenomic analyses of primary high-grade serous ovarian cancers and does not provide sufficient data on chemoresistant and recurrent tumors. Accordingly, the mechanisms underlying chemoresistance and recurrence in ovarian cancers need to be better elucidated to develop improved treatments for this devastating disease.

One of the current emerging concepts concerning tumorigenesis is that tumors are composed of heterogeneous populations of cells with different biological properties and tumorigenic potentials. The cancer stem cell or tumor-initiating cell (CSC/TIC) subpopulation, which has a high tumorigenic potential, is thought to play a crucial role in tumor development, chemoresistance and relapse after initial treatment [4,5]. Since the first identification of a CSC-like population in leukemia, CSC/TICs have been found in ovarian cancer and several solid tumors [4]. Furthermore, this population of cells is thought to be responsible for the high rate of recurrence and chemoresistance of ovarian cancer. Therefore, there is a great need to understand the mechanisms regulating ovarian CSC/TICs in order to develop more effective therapies for ovarian cancer that can overcome the limitations of the currently available therapies.

In this review, we briefly summarize the recent findings regarding genes and signaling pathways that regulate ovarian CSC/TICs, which have helped improve understanding of the mechanisms that contribute to ovarian pathophysiology, including the high level of recurrence and chemoresistance associated with this malignancy. Important microRNAs (miRNAs) and tumor microenvironment features that are responsible for the maintenance and function of ovarian CSC/TICs are also described. Furthermore, current and potential approaches targeting ovarian CSC/TICs will be illustrated to provide insight into future treatment strategies for recurrent ovarian cancer.

## 2. Ovarian CSC/TICs

### 2.1. Concept of CSC/TICs

The concept of CSC/TICs has emerged based on the identification and characterization of a subpopulation of stem-like cells in several distinct malignancies; however, there remain several controversies concerning CSC/TICs. More recently, this concept has evolved into a more comprehensive model that better explains the complex processes involved in tumorigenesis [4]. CSC/TICs are classically defined by the following properties [5]: first, CSC/TICs can produce all types of cells in a tumor, which means that a heterogeneous population of cells, including CSC/TICs and non-CSC/TICs, can be generated by CSC/TICs; second, CSC/TICs have an unlimited self-renewal capacity and can therefore divide indefinitely. Quiescence or slow proliferation, and resistance to conventional chemotherapy have also been suggested to be unique properties of CSC/TICs.

In terms of the origin of CSC/TICs, it is currently thought that CSC/TICs are derived from stem cells or from more differentiated cells that acquire CSC/TIC features through the accumulation of genetic and epigenetic alterations. Intriguingly, recent works have demonstrated that CSC/TIC phenotypes, such as self-renewal and pluripotency, can be acquired by the activation of oncogenic genes or the inactivation of tumor suppressor genes [4]. More importantly, compelling evidence suggests that the epithelial-to-mesenchymal transition (EMT) process promotes the emergence of CSC/TICs [6].

Recent discoveries have helped develop an updated model of CSC/TICs in cancer. This model suggests that CSC/TICs are more dynamic than previously thought, that a tumor can contain multiple genetically diverse CSC/TIC clones, and that the frequency and phenotype of CSC/TICs can vary during tumor progression [4].

### 2.2. Ovarian CSC/TICs

Since Bapat *et al.* first reported that stem-like cells may contribute to ovarian tumorigenesis [7], CSC/TIC populations have been identified in primary ovarian tumors and ovarian cancer cell lines [8]. A recent study further demonstrated the high expression of CSC/TIC markers in freshly isolated cells from ovarian cancer ascites, highlighting the potential enrichment of CSC/TIC population in malignant ascites [9]. Ovarian cancer side populations (Hoechst-excluding cells) have tumorigenic and chemoresistant stem cell-like properties [10–12]. Furthermore, several cell surface markers, including CD44, CD117, CD133, CD24 and aldehyde dehydrogenase-1 A1 (ALDH1 or ALDH1A1), or a combination of these markers, define ovarian CSC/TIC populations in ovarian tumors. Importantly, these markers not only identify ovarian CSC/TICs but are also associated with the clinical outcomes of patients, which suggests that they are promising targets for the treatment of ovarian cancer [13–18]. In addition, there was a recent study reporting that ovarian CSC/TICs isolated from fresh tumor samples of ovarian cancer patients have a mesenchymal phenotype [19]. Interestingly, the repeated treatment of cisplatin in ovarian cancer cell line originated from malignant ascites was also shown to generate a highly chemoresistant subpopulation of cells with stem cell-like features, suggesting that ovarian CSC/TICs can be induced by repeated chemotherapy [20].

#### 2.2.1. CD44, CD117, and CD24

The hyaluronic acid receptor CD44 and the stem cell factor receptor CD117 (c-kit) are cell surface markers of ovarian CSC/TICs. CD44^+^CD117^+^ cells isolated from human ovarian adenocarcinomas are a subpopulation with an ovarian tumor-initiating capacity, which can recapitulate original tumors from which they are derived when injected into mice [21]. CD44^+^ cells, including CD44^+^MyD88^+^ cells from either ascites or tumor tissues [22] and CD44^+^CD24^+^EpCAM^+^ cells from ovarian cancer cell lines [23], exhibit the molecular phenotypes of ovarian CSC/TICs. CD117^+^ ovarian cancer cells exhibit properties unique to CSC/TICs; these properties are associated with self-renewal, high tumorigenic potential, differentiation and chemoresistance of CD117^+^ cells sorted from established mouse xenograft using human ovarian serous adenocarcinoma [24]. Additionally, CD24^+^ cells isolated from patient tumor specimens are enriched in ovarian CSC/TICs and have stem-like properties, including higher levels of chemoresistance, self-renewal and differentiation than CD24^−^ cells [25]. Interestingly, studies reported that CD44^+^CD24^−^ cells from epithelial human ovarian cancer cell lines have properties of ovarian CSCs/TICs [13,26], whereas another study reported that CD24^+^ cells from ovarian cancer patient samples are a subpopulation of ovarian CSC/TICs [25]. A recent study by Meng *et al.* further demonstrated that the CD44^+^CD24^−^ cells found within primary ovarian cancer ascites are associated with an increased risk of recurrence and shorter progression-free survival in patients with ovarian cancer [13].

#### 2.2.2. CD133 and ALDH1

Baba *et al.* first identified CD133 as a marker of ovarian CSC/TICs [27], and further studies [28,29] indicated that CD133 expression can define a tumor-initiating cell population in primary human ovarian tumors. ALDH1 activity was also reported to be associated with a subpopulation of cells with tumor-initiating or CSC properties [16]. Furthermore, more recent studies have indicated that the expression of ALDH1 and CD133 defines a population of ovarian CSC/TICs [18,29]. In addition, CD133 and ALDH1 expression in tumor tissues are associated with the clinical outcomes of ovarian cancer patients, which suggests that they are promising therapeutic targets for ovarian cancer. For instance, CD133 expression in ovarian carcinoma samples is related to a poor prognosis, including shorter overall and disease-free survival [14]. Importantly, this study found that CD133 expression is an independent predictor of poor prognosis, suggesting that CD133 is a useful prognostic factor in ovarian cancer. The clinical significance of ALDH1 was reinforced by current studies, which showed a correlation between high ALDH1 activity in tumor specimens and poor clinical outcome of patients [15–17]. Consistently, the presence of ALDH^+^CD133^+^ cells in debulked primary tumor samples correlates with shorter overall and disease-free survival in ovarian cancer patients [18]. Collectively, these studies indicate that CSC/TICs can affect the clinical outcomes of ovarian cancer patients.

## 3. Genes and Pathways Regulating Ovarian CSC/TICs

### 3.1. Pathways Involved in the Regulation of Ovarian CSC/TICs

Several developmental pathways, including Notch, Wnt, Hedgehog, and transforming growth factor-β (TGF-β), are crucial for the regulation of the self-renewal and maintenance of CSC/TICs [30–32]. Inhibition of these pathways has been suggested as a promising therapeutic strategy, in combination with traditional chemotherapies, for recurrent malignancies. In agreement with the importance of these pathways in the maintenance of CSC/TIC properties, recent studies have reported that these pathways are critical for the regulation of ovarian CSC/TICs, as outlined below.

First, the Notch pathway plays a role in the regulation of ovarian CSC/TICs. Amplification and overexpression of the Notch 3 gene are frequently found in ovarian cancer tissues [33], and Notch 3 expression correlates with poor prognosis of ovarian cancer patients [34]. Recent comprehensive analyses of ovarian serous adenocarcinoma tissues by TCGA further indicated that the Notch pathway is dysregulated in ovarian cancer and is involved in ovarian tumorigenesis [3]. Epigenetic alterations in multiple Notch target genes were clinically significant [35]. This study showed that there is an inverse relationship between epigenetic modifications (DNA methylation and miRNAs) and multiple Notch pathway genes (*PPARG*, *CCND1*, and *RUNX1*) in high-grade serous ovarian cancer tissues. Moreover, patients expressing higher levels of these genes, together with lower levels of DNA methylation in their regions or lower expression levels of their respective miRNAs, had a significantly poorer survival compared with patients with low levels of these genes. In addition to the role of the Notch pathway in ovarian cancer, McAuliffe *et al.* recently suggested that Notch signaling plays a critical role in the maintenance of ovarian CSC/TICs and tumor resistance to platinum [36]. In this study, Notch 3 overexpression increased the ovarian cancer side population, while treatment with a Notch 3 inhibitor depleted the ovarian CSC/TIC population and increased sensitivity to platinum.

Second, the Hedgehog signaling pathway, which is another well-known pathway involved in embryonic development, is implicated in several cancers, including ovarian cancer [30,31]. This pathway is aberrantly activated in ovarian carcinomas, and activation of this pathway affects growth, motility and invasion of ovarian cancer cells [37–39]. Moreover, inhibition of the Hedgehog pathway suppressed growth of an ovarian cancer tumor in a xenograft model, suggesting that this pathway contributes to ovarian tumorigenesis [40]. A recent study reported that the Hedgehog pathway regulates the growth of ovarian cancer spheroid-forming cells, suggesting that this pathway may be involved in the maintenance of ovarian CSC/TICs [41].

Third, EMT is a cellular reorganization process that is essential for embryonic development and also occurs during tumorigenesis, allowing some cancer cells to become more migratory and invasive [30]. Accumulating evidence indicates that CSC/TICs can be generated by EMT [6,42]. TGF-β signaling plays a key role in the modulation of EMT; therefore, it has been suggested that TGF-β signaling is involved in the regulation and maintenance of CSC/TICs. Indeed, there is interplay between ovarian CSC/TICs and EMT [43], and TGF-β signaling is involved in the induction of ovarian CSC/TICs through modulation of its target enzyme [44]. TGF-β-induced tissue transglutaminase (TGM2) promotes ovarian tumor metastasis in association with the induction of EMT and CSC/TIC phenotypes in ovarian cancer [44]. Importantly, ovarian CSC/TICs can be generated by dedifferentiation of ovarian cancer cells.

Lastly, although genetic mutations in the Wnt pathway are rare in epithelial ovarian cancer, recent studies showed that activation of Wnt signaling mediated by the unique ovarian tumor microenvironment may play a role in ovarian tumorigenesis [45]. The factors in the ovarian tumor microenvironment that are involved in the transcriptional activation of Wnt/β-catenin target genes include the interaction of lysophosphatidic acid with its receptor, the interaction of endothelin with its receptor, integrin aggregation and mechanical compression by intraperitoneal fluid pressure. Wnt signaling is also likely to contribute to the maintenance of ovarian CSC/TICs since this signaling pathway is thought to be involved in the regulation of CSC/TICs in other tumors [30,46,47]. In summary, common pathways relevant to the function of CSC/TICs in other tumors possibly also play crucial roles in the regulation of ovarian CSC/TICs.

In addition to the aforementioned stem cell pathways, the NF-κB signaling pathway, which is commonly activated by inflammatory cytokines, affects the survival of ovarian CSC/TICs. NF-κB is constitutively active in ovarian CD44^+^ CSC/TICs, and inhibition of constitutive and TNF-α-induced NF-κB activity results in apoptotic cell death [48]. Similarly, a recent study further demonstrated that toll-like receptor 2 (TLR2) activated pro-inflammatory NF-κB pathway enhances the self-renewal of ovarian CD44^+^MyD88^+^ CSC/TICs, supporting the important role of this pathway in ovarian CSC/TICs [49].

### 3.2. Genes Involved in the Regulation of Ovarian CSC/TICs

Growing evidence suggests that cancer-associated genes play functional roles in the development and maintenance of CSC/TICs, in addition to their roles in ovarian cancer cells. These genes include well-known tumor suppressor genes, CSC/TIC marker genes and EMT-related genes.

First, loss of p53 function, a tumor suppressor protein, is related to the pathogenesis of high-grade serous adenocarcinoma of the ovary [50], and this was further confirmed in the recent TCGA study [3]. In addition, a recent study showed that ovarian CSC/TICs can be generated by depletion of p53 expression in tumor cells [51]. This suggests that p53 dysfunction can enhance the self-renewal ability of ovarian stem-like tumor cells and might thereby contribute to the molecular heterogeneity of ovarian cancers.

Chau *et al.* revealed that c-kit can regulate the survival and proliferation of ovarian CSC/TICs as well as chemoresistance, and its action on ovarian CSC/TICs is mediated by phosphoinositide 3-kinase (PI3K)/Akt and Wnt/β-catenin-ATP-binding cassette G2 (ABCG2) signaling [52]. c-kit is overexpressed in ovarian CSC/TICs and knockdown of c-kit expression or inhibition of c-kit activity by Imatinib (Gleevec) decreases the tumorigenic capabilities and chemoresistance of ovarian CSC/TICs. The effect of c-kit on chemoresistance is associated with its induction of *ABCG2* expression, a multidrug resistance gene. A mechanistic study revealed that c-kit regulates ABCG2 expression through β-catenin. Moreover, this study identified the importance of conditions in the tumor microenvironment, such as hypoxia, in enhancing the survival of ovarian CSC/TICs via upregulation of c-kit expression. In support of previous findings showing that c-kit expression is related to chemotherapy [24], this study might provide a rationale for treatment of recurrent ovarian cancer with a c-kit inhibitor.

Twist is a transcription factor implicated in several processes in human malignancies, including EMT, invasion, survival and drug resistance [53]. A recent study reported that twist-related protein 1 (TWIST1) is involved in the differentiation of ovarian CSC/TICs (type I/CD44^+^ cells) isolated from either ovarian ascites or ovarian cancer tissues [54]. This effect is mediated by positive regulation of miRNAs by TWIST1, including miR-199a and miR-214. In ovarian stem-like cells, TWIST1 expression is undetectable and this results in the low expression of these miRNAs, thereby promoting PTEN and IKKβ activity, which are downstream genes of miR-199a and miR-214. This slows proliferation, blocks apoptosis, and increases inflammation in ovarian CSC/TICs. Conversely, increased TWIST1 expression and a concomitant reduction in PTEN and IKKβ activity was observed in differentiated ovarian cancer cells, indicating that TWIST1 regulates ovarian CSC/TIC differentiation. Differentiated CSC/TIC populations often exhibit chemosensitivity and the inability to undergo self-renewal and repair; therefore, induction of CSC differentiation has been suggested as a possible mechanism for cancer therapy [55]. A recent study indicated that TWIST1 expression is associated with the differentiation of CD44^+^MyD88^+^ ovarian CSC/TICs into cancer cells with mesenchymal features both *in vitro* and *in vivo*, confirming the importance of TWIST1 in the differentiation of ovarian CSC/TICs [56]. Importantly, this study showed that epithelial ovarian CSC/TICs can undergo EMT, and that mesenchymal spheroid-forming cells with metastatic potential can be generated through EMT, suggesting that CSC/TICs contribute to the formation of metastatic ovarian cancer. Furthermore, this study demonstrated that TWIST1 has a crucial role in the induction of EMT in CSC/TICs, while TWIST1 expression is low in ovarian CSC/TICs due to constitutive proteosomal degradation.

The clinical relevance of stem cell-associated genes in ovarian cancer has been uncovered. For instance, Lin-28 is a reprogramming factor that can convert somatic differentiated cells into induced pluripotent stem cells [57]. Recently, it was reported that high expression of the stem cell-associated gene *LIN28B* is related to a poor outcome, including higher mortality and relapse in patients with ovarian cancer, suggesting that this cancer stem cell-associated marker may be a therapeutic target in epithelial ovarian cancer [58]. *NANOG* is another stem cell-associated gene that is associated with ovarian cancer. Homeobox protein NANOG is a stem cell transcription factor that is involved in the maintenance of self-renewal and pluripotency in stem cells. Siu *et al.*[59] investigated the function of NANOG in ovarian tumorigenesis and found that overexpression of NANOG in the nucleus is significantly associated with high-grade serous ovarian cancer, increased chemoresistance, and poorer overall and disease-free survival of patients. Moreover, high NANOG expression is an independent poor prognostic factor. Depletion of NANOG inhibits the proliferation, migration, and invasion of ovarian cancer cells with concomitant increases in the mRNA levels of E-cadherin, caveolin-1, FOXO1, FOXO3a, FOXJ1 and FOXB1 in these cells. Conversely, proliferation, migration and invasion are enhanced in ovarian cancer cells overexpressing NANOG. These effects are mediated by the regulation of E-cadherin and FOXJ by NANOG.

Given that these stem cell-associated genes are involved in the regulation of CSC/TICs in several tumors, it is likely that *LIN28/LIN28B* and *NANOG* regulate ovarian CSC/TICs. In support of this, *LIN28* modulates ADLH1^+^ CSC/TICs and thereby contributes to the maintenance of CSC/TICs [60]. Maintenance of ALDH1^+^ cells by *LIN28* is mediated by let-7, and a *LIN28*/let-7 loop regulates the differentiation and self-renewal of mammary gland epithelial progenitor cells, supporting the idea that a reprogramming-like process generates CSC/TICs. Intriguingly, the involvement of the reprogramming factors Lin-28 and Oct4 in stem-like cells in ovarian cancer was suggested based on the correlation of their coexpression with advanced ovarian cancer tumor grades, and their roles in cell growth and survival [61]. Moreover, NANOG mediates the effects of miR-214 in ovarian CSC/TICs [62]. Taken together, these results suggest that stem cell-associated genes, including *LIN28*, *OCT4,* and *NANOG* play critical roles in the regulation of ovarian CSC/TICs.

Silencing of developmental genes in embryonic stem cells and pluripotent embryonic carcinoma cells by bivalent histone modifications containing active H3K4me3 and repressive H3K27me3 [63,64] was used to identify gene sets associated with high-grade serous ovarian cancer and hence assess the relationship between epigenetic silencing and tumor progression [65]. Novel ovarian cancer-specific bivalent marked genes, which were not observed in embryonic stem cells, were identified, and this gene set was enriched in genes that are modulated by the PI3K and TGF-β signaling pathways. Ovarian tumor “stem cell-like” cells expressed significantly lower levels of bivalent gene sets than matched non-tumor sustaining cells. Furthermore, the expression of these bivalent gene sets was lower in chemoresistant cells than in chemosensitive cells. Thus, these bivalent gene sets are associated with stem cell-like and chemoresistant ovarian cancer cells, meaning that they might include genes that are involved in the regulation of ovarian CSC/TICs. Therefore, further investigation of these bivalent marked genes may help identify key regulators of ovarian CSC/TICs.

## 4. MiRNAs Regulating Ovarian CSC/TICs

MiRNAs have recently emerged as an important regulator of CSC/TICs. In addition to their role in the functions of normal stem cells, including self-renewal and differentiation, miRNAs are involved in tumorigenesis [66]. Furthermore, recent studies indicate that miRNAs play a crucial role in tumor development via the regulation of CSC/TICs.

In most studied breast CSC/TICs, miRNAs such as let-7, miR-30 and miR-200 regulate critical properties of CSC/TICs by targeting their respective downstream genes; for example, let-7 targets *RAS* (self-renewal) and *HMGA2* (differentiation), and miR-200 targets *BMI1* (self-renewal) and *ZEB1/2* (EMT, motility and invasion) [67–71]. Let-7 also modulates ALDH1^+^ CSC/TICs via the regulation of *LIN28* expression and interestingly, *LIN28* is involved in the biogenesis of let-7, suggesting that there is a double-negative feedback loop between *LIN28* and let-7 [60]. Other miRNAs, including miR-451, miR-128, and miR-34a, are reported to be involved in the modulation of glioblastoma multiforme CSCs [72,73]. In particular, miR-34 inhibits the properties of multiple types of CSC/TICs, including prostate [74], pancreatic [75], gastric [76], and glioblastoma multiforme CSC/TICs [73]. MiR-181 affects hepatocellular carcinoma CSC/TICs [77]. Similarly, recent studies have reported that multiple miRNAs also regulate critical features of ovarian CSC/TICs by modulation of CSC/TIC markers or stem cell genes (Table 1).

Cheng *et al.* reported that miR-199a suppresses the tumorigenicity and chemoresistance of ovarian CSCs/TICs (CD44^+^CD117^+^ population) enriched from human primary ovarian tumor tissues [78]. MiR-199a represses CD44 expression by directly binding to the 3′ UTR region of CD44 and thereby inhibits the proliferation, migration and invasion of CD44^+^CD117^+^ ovarian CSC/TICs. Inhibition of CD44 by miR-199a also reduces the mRNA level of the multidrug resistance gene *ABCG2*, and thereby increases the chemosensitivity of ovarian CSCs/TICs. *In vivo* data using xenograft models confirmed the suppression of tumor growth by miR-199a. These observations suggest that miR-199a can suppress ovarian CSCs/TICs by regulating the expression of CD44.

An association between miR-214 and ovarian CSC/TIC properties was demonstrated by a recent study, which found that miR-214 regulates ovarian CSC/TICs in association with p53/NANOG [62]. However, in contrast to the previously reported association of high expression of miR-214 with the differentiation of ovarian CSC/TICs isolated from either ovarian ascites or ovarian cancer tissues [54], overexpression of miR-214 enhanced the sphere growth of ovarian CSC/TIC *in vitro*, whereas depletion of miR-214 expression decreased the number of the ovarian CSC/TIC population and the self-renewal capacity of these cells. These effects are associated with increased expression of *NANOG* due to miR-214. MiR-214 represses p53 by directly binding to the 3′ UTR of the p53 gene, and this increases the level of NANOG mRNA because NANOG is transcriptionally repressed by p53. This is in agreement with a previous study that demonstrated a link between the depletion of p53 and the expansion of ovarian CSC/TICs [51].

MiR-200a is also involved in the regulation of ovarian CSC/TICs. In ovarian cancer cell lines, miR-200a is downregulated in CD133^+^ cells in comparison to its level in CD133^−^ cells. Overexpression of miR-200a reduces the migration and invasion of CD133^+^ ovarian CSC/TICs, suggesting that miR-200a functionally inhibits ovarian CSC/TICs [79]. Moreover, this study indicated that inhibition of CD133^+^ ovarian CSC/TICs is related to the targeting of *ZEB2* by miR-200a.

Additionally, profiling of dysregulated miRNA expression in the CD133^+^ subpopulation of ovarian cancer cell line indicated that several miRNAs are upregulated, including miR-205, miR-146a, miR-200a and miR-200b, whereas miR-1202 and miR-1181 are downregulated, which indicates that miRNAs can modulate the properties of ovarian CSC/TICs [80]. Further studies are required to confirm the functional roles of these miRNAs in ovarian CSC/TICs.

Consistent with the effect of let-7 on tumorigenesis in other tumor types, the let-7 pathway is deregulated in a specific molecular subtype of high-grade serous ovarian cancer tissues [81]. Activation of this oncogenic pathway, which involves *MYCN*, *LIN28B,* and *HMGA2*, represses let-7 and consequently the let-7 target gene *HMGA2* is overexpressed. An association between let-7 and its target genes has not yet been demonstrated in ovarian CSC/TICs; however, given the roles of *LIN28B* and *HMGA2* in CSC/TICs, it is possible that these genes and other stem cell genes related to the let-7 pathway play a role in the differentiation of ovarian CSC/TICs. Further studies are required to fully elucidate the role of the let-7 pathway in the regulation of ovarian CSC/TICs.

It is noteworthy that the aforementioned miRNAs, including miR-214, miR-200 and let-7, also play important roles in ovarian cancer cells [82]. This suggests that targeting of these miRNAs may be an effective therapeutic strategy for targeting both ovarian CSC/TICs and cancer cells.

## 5. Regulation of Ovarian CSC/TICs by the Tumor Microenvironment

**Figure 1:**
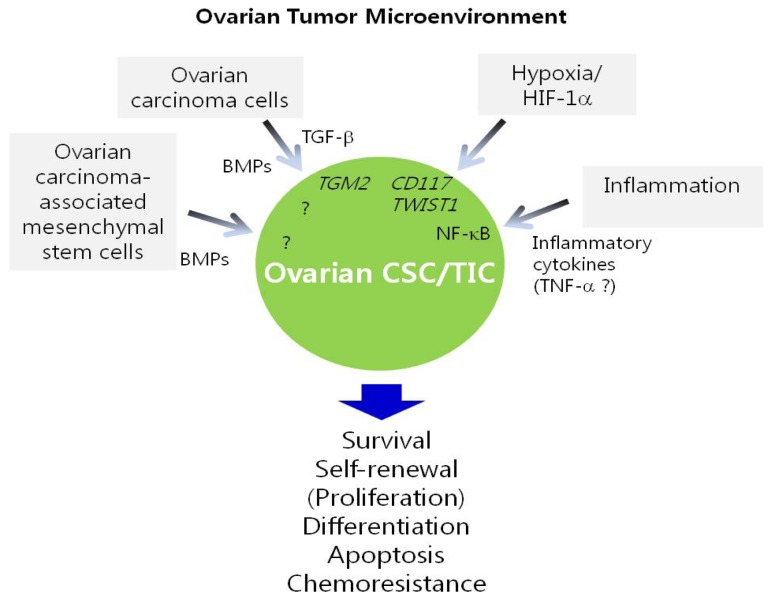
The tumor microenvironment involved in the maintenance and regulation of ovarian CSC/TICs. The maintenance and properties of the ovarian CSC/TIC population are influenced by the surrounding microenvironment, termed the “CSC/TIC niche.” Growth factors, such as bone morphogenetic protein (BMP) and transforming growth factor-β (TGF-β) released from ovarian cancer cells, increase the self-renewal and proliferation of CSC/TICs. Ovarian carcinoma associated-mesenchymal stem cells (CA-MSCs) are also reported to regulate ovarian CSC/TICs through BMP signaling. Moreover, hypoxia, which is a common feature of the ovarian cancer microenvironment, enhances the survival and chemoresistance of ovarian CSC/TICs through the upregulation of c-kit mediated by HIF-1α. Conversely, hypoxic induction of HIF-1 expression can promote the differentiation of ovarian CSC/TICs by inducing TWIST1 expression. A high level of NF-κB activity in ovarian CSC/TICs may be induced by cytokines, such as TNF-α, that are generated as a result of inflammation in the ovarian tumor microenvironment.

As discussed above, a variety of genes, pathways, and miRNAs are implicated in the regulation of ovarian CSC/TICs. In addition, CSC/TIC traits, for example being undifferentiated and the capacity for self-renewal, might be maintained by their surrounding environment, termed the “CSC/TIC niche” [83]. This niche is a microenvironment composed of heterogeneous populations, including diverse immune cells, stromal cells, blood vessels and extracellular matrix components [84]. Signaling from this microenvironment can activate pathways required for the maintenance and function of CSC/TICs. Although understanding of this unique microenvironment is limited, emerging evidence indicates that the CSC/TIC niche is important for sustaining CSC/TICs. More importantly, it has been suggested that differentiated cells can revert to CSC/TICs and, conversely, CSC/TICs can differentiate into non-CSC/TICs in specific tumor microenvironments, which highlights the crucial role of the tumor microenvironment in the emergence of CSC/TICs [83]. Several biological processes that occur in the CSC/TIC niche, including EMT, inflammation, hypoxia and angiogenesis, are believed to determine the fate of CSC/TICs [83]. It is being increasingly reported that the tumor microenvironment contributes to the function of ovarian CSC/TICs (Figure 1).

Several recent studies have reported that hypoxia is one of the key attributes of the tumor microenvironment that regulates the properties of ovarian CSC/TICs. For instance, hypoxia increases the survival and chemoresistance of ovarian CSC/TICs isolated from ovarian cancer cell lines through the induction of c-kit expression mediated by HIF-1α [52]. In CD44^+^MyD88^+^ ovarian CSC/TICs from either ovarian cancer tissues or ovarian ascites, hypoxia/HIF-1 is an extracellular signal that may initiate differentiation of CSC/TICs by triggering TWIST1 expression [56]. Under hypoxic conditions, ovarian cancer cells acquire stem-like properties through the upregulation of stemness-associated genes such as *OCT3/4* and *SOX2*[85], further supporting the link between a hypoxic microenvironment and the properties of ovarian CSC/TICs.

TGF-β that is secreted into the ovarian tumor microenvironment also affects the growth of ovarian CSC/TICs [44]. Moreover, secreted TGF-β in ovarian cancer cells promotes ovarian tumor metastasis by inducing EMT and stem-like cells [44].

Importantly, McLean *et al.* reported that ovarian carcinoma associated-mesenchymal stem cells (CA-MSCs) within the ovarian tumor microenvironment promote tumor growth by increasing the number of CSC/TICs [86]. The effects of CA-MSCs on CSC/TICs are mediated in part through the bone morphogenetic protein (BMP) signaling pathway, highlighting BMP signaling as a potential therapeutic target in ovarian cancers. Lin-28 is reported to regulate BMP4 in ovarian cancer [87]; this study hypothesized that BMP4, a growth factor that is highly produced in ovarian cancer cells, or BMPs derived from ovarian CA-MSCs, may stimulate CSC/TIC proliferation and thereby promote tumor growth.

Cancer-associated fibroblasts (CAFs) are a type of stromal cells that constitute the tumor microenvironment and promote tumorigenesis by enhancing cell proliferation, angiogenesis, invasion and metastasis [84]. CAFs enhance the sphere formation of prostate CSC/TICs, suggesting that CAFs regulate CSC/TICs [88]. It is possible that ovarian CSC/TICs might also be influenced by CAFs, although this remains to be confirmed. Interestingly, a recent study reported that the expression levels of specific miRNAs, including miR-31, miR-214, and miR-155, differ between normal ovarian tissue fibroblasts and ovarian CAFs [89]. Notably, this study demonstrated that the manipulation of miRNAs can convert normal ovarian fibroblasts into ovarian CAFs and vice versa. This indicates that ovarian cancer cells can reprogram stromal cells through the regulation of miRNAs, and suggests that the targeting of miRNAs in stromal cells may be effective for the treatment of ovarian cancer.

## 6. Genes and Pathways Involved in Chemoresistance and Recurrence of Ovarian Cancer

The main focus of ovarian cancer therapy is to overcome chemoresistance and recurrence after initial therapy since these are the major causes of mortality in ovarian cancer patients. Chemoresistance can be classified into two major forms: “de novo or inherent” and “acquired,” both of which are ultimately linked to disease recurrence [90]. Patients may be refractory to initial therapy, or may relapse due to the acquirement of resistance after initial therapy, even though they were sensitive to the initial therapy. Accumulating evidence indicates that the CSC/TIC population within tumors is responsible for the acquisition of chemoresistance and ultimately disease recurrence. CSC/TICs are thought to be resistant to traditional cytotoxic anti-cancer drugs due to their unique properties [4,91]. For instance, decreased responsiveness to chemotherapy might be due in part to the slow proliferation rates of CSC/TICs given that conventional cytotoxic drugs mainly target highly proliferative cells. Other features of CSC/TICs that have been suggested to be responsible for chemoresistance include high expression of ATP-binding cassette drug transporters and anti-apoptotic proteins, the ability to protect cells from DNA damage, and efficient DNA repair [91]. In agreement with CSC/TICs being responsible for chemoresistance, several studies have demonstrated that CSC/TICs are more resistant to chemotherapy than non-CSC/TICs, and that cells with CSC/TIC markers persist after chemotherapy, whereas cells lacking CSC/TIC markers are eliminated [5].

Several studies have explored the mechanisms underlying chemoresistance in ovarian cancer. Steg *et al.* sought to elucidate the CSC/TIC-related pathways responsible for clinical chemoresistance in ovarian cancer [90]. When matched primary and recurrent tumor samples were examined, populations positive for ovarian CSC/TIC markers, such as ALDHA1, CD44 and CD133 increased after primary therapy, while the percentage of cells positive for each marker was similar in primary tumors and tumors examined at first recurrence before secondary therapy. Notably, of the CSC/TIC markers, only CD133 expression was significantly higher in tumors from recurrent and chemoresistant patients than in primary tumors. Moreover, the expression of 12 of the 84 CSC/TIC pathway-related genes was significantly higher in recurrent tumors than in matched primary tumors. These genes include members of the TGF-β (*ENG*, *ZEB2*, *LTBP4*, *TGFBR2*, *RGMA*, *ACVR1B*, and *SMAD2*), Hedgehog (*GLI1* and *GLI2*), Notch (*PSEN2*) and Wnt (*FZD9* and *BCL9L*) pathways. Specifically, the TGF-β co-receptor endoglin (CD105) and the Hedgehog mediator Gli 1/2, which are overexpressed in recurrent ovarian cancers, both affect the viability of ovarian cancer cells, and Gli 2 also affects resistance to cisplatin. These findings indicate that the CSC/TIC subpopulation is enriched in tumors after primary therapy and that targeting CSC/TIC pathway genes may overcome clinical chemoresistance.

Similarly, it was recently reported that endoglin, a member of TGF-β pathway, is involved in ovarian chemoresistance [92]. Downregulation of endoglin enhances apoptosis, induces DNA damage, increases sensitivity to cisplatin, and decreases cell viability. Specifically, DNA damage induced by the inhibition of endoglin was associated with the inhibition of several DNA repair genes, such as *BARD1*, *H2AFX*, *NBN*, *NTHL1*, and *SIRT1*. As this membrane protein is highly expressed in ovarian chemoresistant CSC/TICs and tumor-associated endothelial cells, inhibiting endoglin might increase platinum sensitivity and be effective for the treatment of ovarian cancer by targeting both tumor angiogenesis and ovarian CSC/TICs.

On the basis of the involvement of Notch signaling in the chemoresistance of ovarian cancer, a recent study [36] demonstrated that the Notch signaling pathway, specifically Notch 3, is critical for platinum resistance in ovarian CSC/TICs. Treatment with a Notch pathway inhibitor increases the sensitivity of ovarian cancer to cisplatin and reduces the ovarian CSC/TIC population. Moreover, knockdown of Notch 3 enhances the response to cisplatin. This study provides evidence of a synergic response when combination therapy of cisplatin and a Notch 3 inhibitor are used. Importantly, treatment with a Notch 3 inhibitor enhances the cisplatin response by augmenting the DNA damage and cell death caused by cisplatin. This suggests that a combination of cisplatin and Notch 3 inhibitor treatments target both CSC/TIC and non-CSC/TIC bulk tumor cells.

In addition, the ovarian CSC/TIC marker c-kit is involved in chemoresistance in ovarian cancer cells by regulating ABCG2 through β-catenin [52]. Depletion of c-kit reduces the chemoresistance of ovarian CSC/TICs.

## 7. Therapeutic Targeting of Ovarian CSC/TICs

The current approach of anti-cancer drug discovery has shifted from conventional cytotoxic drugs to molecular targeted therapy. High-throughput approaches have identified numerous potential therapeutic targets specific to cancer cells. However, this approach has focused on the killing of bulk tumor cells rather than targeting CSC/TICs.

The current failures in cancer therapy are mostly due to tumor recurrence after therapy and not to a defective response to initial therapy. A major challenge now is to discover agents and strategies that eliminate the sources of tumor recurrence and chemoresistance along with bulk cancer cells. As discussed above, CSC/TICs have crucial roles in tumor recurrence after therapy; therefore, targeting CSC/TICs has been suggested as a promising way to achieve a long-term cure for cancer without further recurrence.

Progression has been made in developing strategies that target CSC/TICs. Recent searches for therapeutic agents and drugs that efficiently eradicate CSC/TICs can be classified into two main approaches: (1) the identification of agents that inhibit CSC/TIC markers or CSC/TIC-specific pathways believed to be critical for the regulation of CSC/TIC properties [91], and (2) high-throughput screening of CSC/TIC-enriched populations with a number of potential drugs [4].

Similar approaches have been used to identify therapeutic agents against ovarian CSC/TICs; such agents deplete the CSC/TIC population, inhibit CSC/TIC growth, or induce CSC/TIC death, and thereby have anti-tumor activity. These agents may be useful in the treatment of patients with recurrent ovarian cancer; however, these studies are at an early experimental stage and further validation is required (Table 2).

The targeting of CSC/TIC markers, such as CD44, CD123, EpCAM, and ATP-binding cassette transporters have been explored as a means of eliminating CSC/TICs [4,91]. Similarly, targeting CSC/TIC markers can inhibit ovarian CSC/TICs. The interaction between the CSC marker CD44 and its ligand hyaluronan are essential for the properties of ovarian CSC/TICs, and antagonization of this interaction by hyaluronan oligosaccharides effectively eliminates highly tumorigenic cells [93]. Claudin-4 is highly expressed in CD44^+^ ovarian CSCs; therefore, the response of ovarian CSC/TICs to clostridium perfringens enterotoxin (CPE) treatment was examined. CPE is cytotoxic to chemoresistant CD44^+^CSC/TICs [94], and CPE administration suppresses tumor progression in mice harboring CD44^+^ CSC/TICs-derived tumors.

With regards to targeting pathways relating to CSC/TICs, treatment with γ-secretase inhibitors, which target the Notch pathway [36], and depletion of tissue transglutaminase, which is involved in the TGF-β pathway [44], decrease the number of ovarian CSC/TICs. Treatment with an inhibitor of the Hedgehog pathway was recently reported to suppress ovarian cancer growth *in vivo*, suggesting that this pathway is a promising molecular target [40].

Based on previous successful identification of CSC/TIC-specific inhibitors in breast cancer [98] and glioblastoma [99], high-throughput screening of ovarian CSC/TIC populations has been used to identify novel drugs that may effectively treat drug-resistant ovarian cancer. Mezencev *et al.* identified a number of candidate ovarian CSC/TIC inhibitors among the 825 compounds from the NCI mechanistic set of the developmental therapeutics program (NCI/NIH) [100]. Among more than 1200 clinically approved drugs, antihelmintic niclosamide was confirmed to selectively target ovarian CSC/TICs [95]. The effects of niclosamide on ovarian CSC/TICs are associated with the inhibition of metabolic pathways related to redox regulation and biogenesis. The effects of these candidate drugs need to be further validated, and this may lead to the discovery of more effective ovarian CSC/TIC inhibitors.

More recently, novel attempts targeting the unique properties of CSC/TICs that are distinct from those of non-CSC/TICs was also used to identify ovarian CSC/TIC-specific inhibitors. Alvero *et al.*[96] identified the isoflavane derivative NV-128 as a compound that targets ovarian CSC/TICs based on the resistance of ovarian CSC/TICs to apoptotic cell death induced by traditional chemotherapeutic agents. Treatment of chemoresistant CD44^+^MyD88^+^ cells with NV-128 induced cell death by inhibiting mitochondrial function and thereby activating cell death pathways. This study showed that inducing cell death in chemoresistant CSC/TIC populations by targeting mitochondria may be a promising approach to treating ovarian cancer. Similarly, a recent study reported that ovarian CSC/TIC-like cells are sensitive to 3-bromopyruvate (3-BP), whose main mechanism of antitumor effect is related to the inhibition of a survival promoting mitochondrial complex, suggesting the use of 3-BP in targeting ovarian CSC/TIC population [20]. A unique characteristic of ovarian CSC/TICs is constitutive NF-κB activity; this was used to identify the natural product, eriocalyxin B, as a potential ovarian cancer treatment. Ericalyxin B induces cell death in ovarian CSC/TIC populations by inhibiting NF-κB activity [48].

As mentioned above, targeting of CSC/TICs has been reported to successfully suppress tumor progression, which supports the belief that CSC/TIC-targeting therapies are promising anti-cancer strategies. However, CSC/TICs may be generated from non-CSC/TICs. This means that CSC/TIC-targeting therapies may eliminate CSC/TICs, but further CSC/TICs may be generated from non-CSC/TICs, leading to incomplete regression of tumors [98]. To address this concern, most current attempts are searching for agents that target both CSC/TICs and non-CSC/TICs in tumors. Moreover, emerging evidence suggests that combination therapies that include agents that are toxic to CSC/TICs and agents that affect non-CSC/TICs, including conventional chemotherapy, are most effective in the treatment of cancer.

Metformin, which is clinically used as an anti-diabetic drug, selectively targets CSC/TICs [97,101] and recent studies have reported the synergic effects of metformin together with chemotherapy or monoclonal antibody therapy in the treatment of multiple types of cancer including breast, prostate and lung. In a mouse xenograft model, combination treatment of metformin with chemotherapeutic agents including doxorubicin, paclitaxel and carboplatin more effectively inhibited tumor growth and prevented relapse than treatment with either drug alone [101,102]. Additionally, concurrent treatment of metformin with an anti-HER2 antibody (trastuzumab) synergically reduced the percentage of trastuzumab-resistant CSC/TICs in breast cancer, indicating that metformin can eradicate the trastuzumab-refractory CSC/TIC population in HER2^+^ breast cancer [103]. Similarly, metformin also targets ovarian CSC/TICs. This drug reduced the number and sphere-forming ability of ALDH^+^ CSC/TICs *in vitro*, and reduced the growth of ALDH^+^ CSC/TICs in xenografts [97]. Notably, metformin also has anti-proliferative and pro-apoptotic effects on ovarian cancer cells *in vitro*[104,105]. More importantly, metformin not only suppresses the angiogenesis and metastasis of ovarian cancer but also enhances cytotoxicity to cisplatin *in vivo*[106]. These findings reveal that metformin targets both ovarian CSC/TICs and non-CSC/TICs, which suggests that this drug is an effective treatment for recurrent ovarian cancer. Although the synergic effect of metformin treatment together with chemotherapeutic agents or therapeutic antibodies has not yet been proven in ovarian cancer, it is possible that combination treatment with metformin and other anti-cancer drugs may be a promising approach to treat recurrent ovarian cancer by targeting both ovarian CSC/TICs and bulk tumor cells. Intriguingly, most of the compounds identified by high-throughput screening of the 825 compounds in the NCI/NIH were shown to inhibit the growth of more differentiated ovarian cancer cells as well as that of ovarian CSC/TICs [100].

It has been suggested that combination treatment of retinoids with platinum therapy is likely to affect both ovarian cancer cells and ovarian CSC/TICs, whereas carboplatin treatment alone does not [107]. Combination treatment of a Notch pathway inhibitor with cisplatin more effectively eliminates CSC/TICs and tumor cells than either treatment alone [36]. Importantly, this combination treatment increase the sensitivity of tumors to cisplatin, thereby promoting synergic cytotoxic effects by enhancing DNA damage, G2/M cell cycle arrest, and cell death.

Since accumulating evidence suggests that the tumor microenvironment plays crucial roles in the regulation of CSC/TICs, targeting of the CSC/TIC niche should be considered in the development of anti-cancer therapies in ovarian cancer.

**Figure 2:**
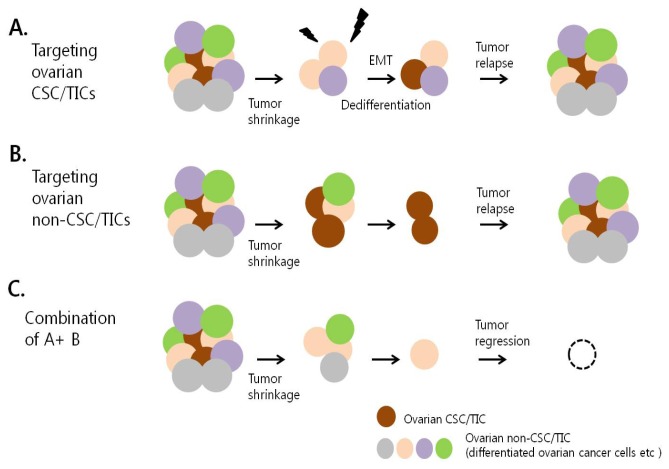
Strategies for the effective treatment of recurrent ovarian cancer. (**A**) Therapeutic strategy to target ovarian CSC/TICs. Therapy targeting ovarian CSC/TICs might eliminate this population from the bulk tumor. However, current studies suggest that differentiated non-CSC/TICs can dedifferentiate into CSC/TICs by EMT or in response to signals from the tumor microenvironment. Therefore, despite initial eradication of the CSC/TIC population, tumor relapse might occur due to the generation of CSC/TICs from the remaining non-CSC/TICs; (**B**) Therapy targeting ovarian non-CSC/TICs and conventional chemotherapy. Conventional cytotoxic chemotherapy and therapies that do not target ovarian CSC/TICs kill most tumor cells but do not remove CSC/TICs from the bulk tumor. Therefore, the tumor initially shrinks but then grows back due to the remaining CSC/TICs; (**C**) Combination therapy targeting both ovarian CSC/TICs and bulk tumor cells. Combination treatment of conventional cytotoxic drugs and other drugs that target ovarian CSC/TICs will lead to tumor degeneration through the complete eradication of CSC/TICs. This therapeutic strategy is postulated to prevent tumor recurrence and allow ovarian cancer to be cured.

Taken together, ovarian tumors are composed of heterogeneous populations, including CSC/TICs, cancer cells and various cells in the tumor microenvironment, including stromal cells. Although CSC/TICs are currently thought to be the main cause of tumor recurrence and chemoresistance, targeting of CSC/TICs alone might not provide the ultimate cure for ovarian cancer since CSC/TICs appear to be induced or emerge from differentiated non-CSC/TICs in response to signals from the tumor microenvironment. Accordingly, targeting both the bulk tumor, including the tumor microenvironment, and CSC/TICs may provide a more effective means of overcoming tumor recurrence and thereby provide a cure for ovarian cancer (Figure 2).

## 8. Conclusions

Growing evidence supporting the existence of ovarian CSC/TICs and their crucial roles in the recurrence and chemoresistance of ovarian cancer has helped identify more effective therapeutic strategies for treating recurrent ovarian cancer. Recent studies have begun to report the genes, pathways, and miRNAs that regulate ovarian CSC/TICs. However, further studies are required to fully understand how the properties of ovarian CSC/TICs are regulated in order to assist the development of therapeutic strategies that target ovarian CSC/TICs. Notably, current evidence suggests that the microenvironment in which CSC/TIC reside, the so-called “CSC/TIC niche,” is pivotal to the maintenance of CSC/TICs; therefore, how the tumor microenvironment regulates CSC/TICs should be considered. A comprehensive understanding of how ovarian CSC/TICs are regulated will allow novel anti-cancer drugs targeting CSC/TICs to be identified. Ultimately, this will facilitate the development of better treatments for recurrent ovarian cancer using combinations of drugs that target both CSC/TICs and non-CSC/TICs in tumors and the tumor microenvironment.

## Figures and Tables

**Table 1:** Genes, pathways, and miRNAs involved in the regulation of ovarian cancer stem cells or tumor-initiating cells (CSC/TICs).

Gene symbol	Gene name or target gene name	Types of ovarian CSC/TICs	Function	Reference
**Genes or pathways (target gene)**				
Notch signaling (*NOTCH3*)	Notch 3	Side population	Expansion of ovarian CSC/TIC population Resistance to platinum	[36]
Hedgehog signaling			Growth of ovarian cancer spheroid-forming cells	[41]
TGF-β signaling (*TGM2*)	Transglutaminase 2 (C polypeptide, protein-glutamine-gamma-glut amyltransferase)	CD44^+^CD117^+^ cells	Self-renewal and expansion of ovarian CSC/TIC population	[44]
NF-κB signaling		CD44^+^ cells	Survival (apoptosis)	[48]
TLR2-MyD88-NF-κB signaling		CD44^+^MyD88^+^ cells	Self-renewal	[49]
*KIT*(*c-kit*, *CD117*)	v-kit Hardy-Zuckerman 4 feline sarcoma viral oncogene homolog		Self-renewal and expansion of ovarian CSC/TICs Resistance to chemotherapy	[52]
*TP53*	p53	EpCAM^+^ cells	Expansion of ovarian CSC/TIC population	[51]
*LIN28*	Lin-28 homolog (*C. elegans*)	ALDH^+^ cells	Expansion of ovarian CSC/TIC population	[60]
*TWIST1*	Twist basic helix-loop-helix transcription factor 1	CD44^+^MyD88^+^ cells	Differentiation	[56]
**miRNAs (target gene)**				
miR-214 (*TP53*)	Tumor protein p53	ALDH1^+^ cells	Self-renewal and expansion of ovarian CSC/TIC population	[62]
miR-199a (*CD44*)	CD44 molecule	CD44^+^CD117^+^ cells	Proliferation and invasion Resistance to chemotherapy	[78]
miR-200a (*ZEB2*)	Zinc finger E-box binding homeobox 2	CD133^+^ cells	Migration and invasion	[79]

**Table 2:** Agents and drugs targeting ovarian CSC/TICs.

Agent or drug	Ovarian CSC/TICs targeted	Target	Effects	Mechanism of action	Reference
**Targeting markers and stem cell pathways related to ovarian CSC/TICs**					
Small hyaluronan oligosaccharides	Cells highly expressing CD133	Hyaluronan-CD44 interaction	Inhibition of growth of ovarian carcinomas with high levels of CD133 *in vivo*	Inhibition (dissociation) of the hyaluronan-CD44 interaction	[93]
Clostridium perfringens enterotoxin (CPE)	CD44^+^ cells	Claudin-4 in CD44^+^ ovarian CSC/TICs	Inhibition of tumor progression of mice harboring xenografts of chemotherapy-resistant CD44^+^ ovarian CSC/TCS *in vivo*	CPE-induced cytotoxicity	[94]
γ-secretase inhibitors	Side population	Notch pathway	Increased sensitivity to cisplatin	Depletion of CSC/TICs	[36]
**Targeting the function and properties of ovarian CSC/TICs**					
Niclosamide	Side population	Metabolic pathways	Inhibition of ovarian CSC/TIC growth *in vitro* and *in vivo*	Disruption of multiple metabolic pathways	[95]
Isoflavane derivate, NV-128	CD44^+^MyD88^+^ cells	Mitochondria (Mitochondrial bioenergetics)	Induction of apoptosis in ovarian CSC/TICs *in vitro*	Inhibition of mitochondrial function	[96]
Eriocalyxin B	CD44^+^ cells	NF-κB pathway	Induction of apoptosis in ovarian CSC/TICs *in vitro*	Inhibition of the NF-κB pathway	[48]
Metformin	ALDH^+^ cells		Inhibition of ovarian CSC/TIC growth *in vitro* and *in vivo*	Depletion of CSC/TICs, and inhibition of the formation of CSC/TIC spheres	[97]
